# Geostatistical interpolation model selection based on ArcGIS and spatio-temporal variability analysis of groundwater level in piedmont plains, northwest China

**DOI:** 10.1186/s40064-016-2073-0

**Published:** 2016-04-11

**Authors:** Yong Xiao, Xiaomin Gu, Shiyang Yin, Jingli Shao, Yali Cui, Qiulan Zhang, Yong Niu

**Affiliations:** School of Water Resources and Environment, China University of Geosciences (Beijing), Beijing, 100083 PR China; Beijing Water Science and Technology Institute, Beijing, 100044 PR China; Shandong Agricultural University, Taian, 271018 PR China

**Keywords:** Groundwater level, Interpolation model, Spatio-temporal variability, Piedmont plain, China

## Abstract

Based on the geo-statistical theory and ArcGIS geo-statistical module, datas of 30 groundwater level observation wells were used to estimate the decline of groundwater level in Beijing piedmont. Seven different interpolation methods (inverse distance weighted interpolation, global polynomial interpolation, local polynomial interpolation, tension spline interpolation, ordinary Kriging interpolation, simple Kriging interpolation and universal Kriging interpolation) were used for interpolating groundwater level between 2001 and 2013. Cross-validation, absolute error and coefficient of determination (R^2^) was applied to evaluate the accuracy of different methods. The result shows that simple Kriging method gave the best fit. The analysis of spatial and temporal variability suggest that the nugget effects from 2001 to 2013 were increasing, which means the spatial correlation weakened gradually under the influence of human activities. The spatial variability in the middle areas of the alluvial–proluvial fan is relatively higher than area in top and bottom. Since the changes of the land use, groundwater level also has a temporal variation, the average decline rate of groundwater level between 2007 and 2013 increases compared with 2001–2006. Urban development and population growth cause over-exploitation of residential and industrial areas. The decline rate of the groundwater level in residential, industrial and river areas is relatively high, while the decreasing of farmland area and development of water-saving irrigation reduce the quantity of water using by agriculture and decline rate of groundwater level in agricultural area is not significant.

## Background

Scarcity of water has become an important issue worldwide (Kahil et al. 2014; Solomon 2015; Karmegam et al. 2010). As the major irrigation water source in arid and semiarid regions, groundwater is an important water source for the development of human society (Smedema and Shiati 2002). While over-exploitation causes a continuous decline in groundwater level. To estimate the degree of overexploitation, the surface trend of groundwater should be known, which can be determined by the available well data integrated in various interpolating methods. Therefore, selecting the best method to estimate the temporal and spatial variations of groundwater level has an important strategic significance for reasonable groundwater management and sustainable development of water resources.

Spatial interpolation is a method to estimate the data in contiguous area and forecast the unknown points (the information is missing or cannot be obtained) with available observation data (Chai et al. 2011; Losser et al. 2014), including geo-statistical interpolation and deterministic interpolation. Geo-statistical interpolation consists of ordinary Kriging interpolation (OK), simple Kriging interpolation (SK) and universal Kriging interpolation (UK); deterministic interpolation comprise global polynomial interpolation (GPI), local polynomial interpolation [inverse distance weighted interpolation (IDW), planar spline interpolation and local polynomial interpolation (LPI)].

The geo-statistical interpolation, or local space interpolation, is known as the unbiased optimal estimation method for describing regionalized variables (Gundogdu and Guney 2007), but has some defects in smooth effects. The local errors can be corrected without reducing the accuracy by posteriori method (Yamamoto 2005); GPI can analyze surface trend of regionalized variables, test the long-term and global trend effect. Based on the sample data, GPI is susceptible to extreme values and is suitable for small surface changes of regionalized variables (Ding et al. 2011; Mutua 2012; Wang et al. 2014); LPI can be used to establish smooth surface, calculate short-term variability and reflect local variability, but has errors for long-distance global interpolation; weight principle is used by IDW for interpolation analysis, as an accurate interpolation method (Xie et al. 2011), it requires sufficient and well-distributed samples (Rabah et al. 2011). Uneven distribution or outliers may lead to errors; tension spline interpolation (TSPLINE) is an accurate interpolation method (Hofierka et al. 2002) and the interpolation surface goes through every sample point, which is suitable for a large number of data interpolation. The accuracy of which is close to Kriging methods. TSPLINE has an advantage to avoid estimation of covariance function structure.

Interpolation methods have been widely used to analyze spatial variability of precipitation, evapotranspiration, temperature and groundwater level by comparing different interpolation methods. Cross-validation along with applicable conditions of different models are applied to obtain the best-fit interpolation model (Mardikis et al. 2005; Yang et al. 2011). With the development of GIS technology, interpolation analysis of groundwater level with geo-statistical modules has become easer and more operable (Salah 2009; Ta’any et al. 2009; Nikroo et al. 2010), which can characterize the spatial variability of variables in detail (Uyan and Cay 2013; Triki et al. 2013; Dinka et al. 2013; Bao et al. 2014).

Geo-statistical method is a good way for analyzing spatial variability of groundwater level by summarizing the previous researches. Based on seven interpolation methods and semi-variable function model in GIS geo-statistical module, the purposes of the study are (1) compare the prediction accuracies of different methods and select the best-fit interpolation model for piedmont plain area; analyze the spatial variability of the groundwater level in piedmont plain based on hydrogeological conditions and semi-variable function, (2) identify the spatial variability characteristics of different hydrogeological units, carry out groundwater level variability partitions considering the land use of piedmont plain and discuss effects of different land use on spatial variability characteristics of groundwater level.

## Research method

### Survey of study area

**Fig. 1:**
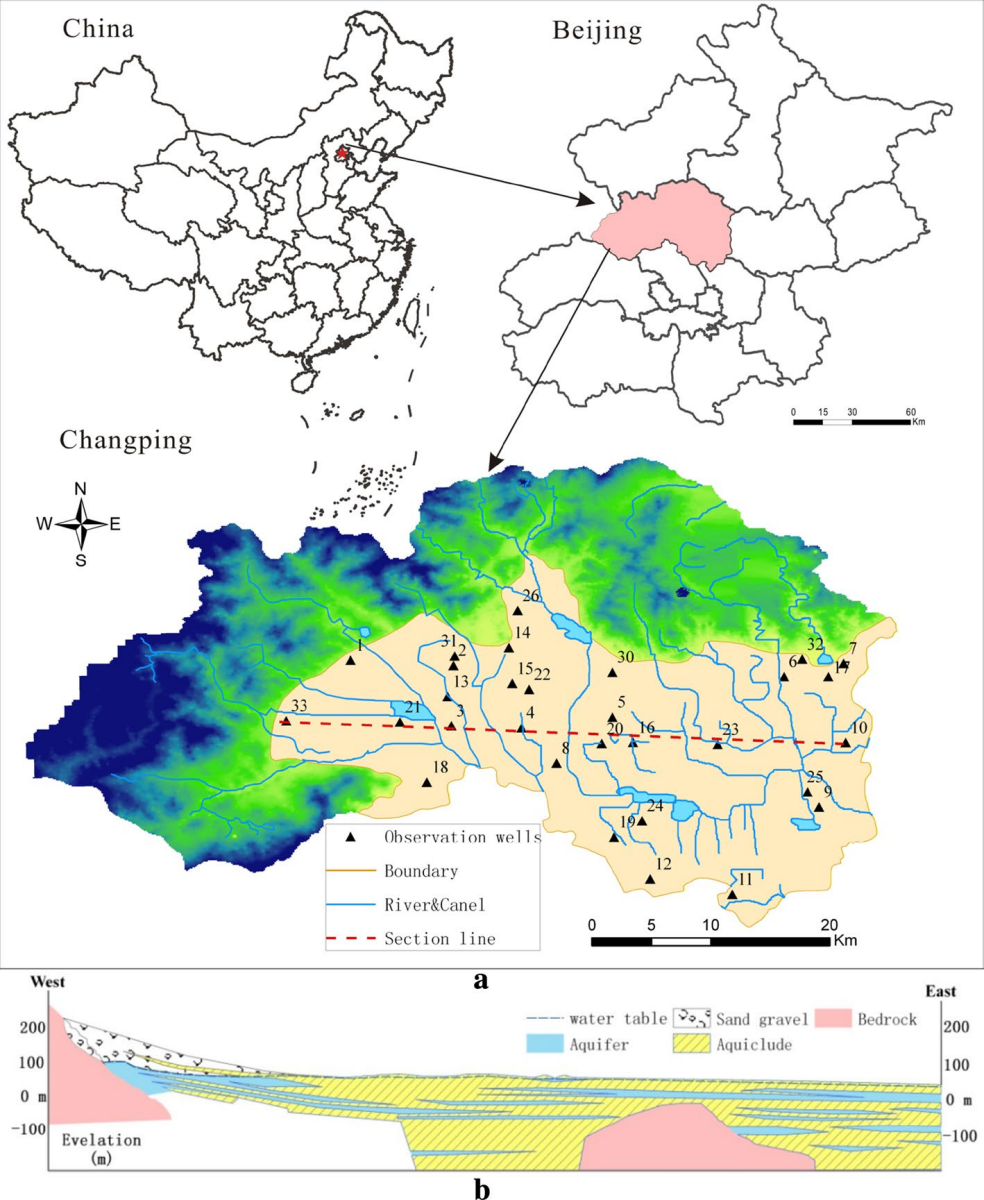
Location of the study area and observation wells (**a**) and hydrogeological map (**b**)

As shown in Fig. 1a, the study area is located in the piedmont plain in west of Beijing and covers an area of 550 km^2^. The topography is high in the northwest and low in the southeast (the elevation is between 30 and 100 m). Owing to the influence of continental monsoon climate, the spatial and temporal distribution of the precipitation is uneven, the mean annual precipitation is 551.4 mm. The piedmont plain is the place where the mountainous area transitions to the plain and the topographic slope is between 1 and 3 %, the aquifer is mainly composed of clay, spall and gravel, the permeability is strong. While the lithological in plain mainly comprises clay and silty, covered by quaternary sediments (Fig. 1b).

The land is mainly used for agriculture. The agricultural area, residential area, industrial area, forest area and the river cover 35.1, 20.5, 10.3, 26.2 and 6.9 % respectively. The water system in this area belongs to Wenyu River basin of the north canal river system. The groundwater regimes type is infiltration—exploitation. Groundwater level shows a seasonal change trend during the year. The drawdown of groundwater level during 2001 and 2013 reaches 22 m and the annual speed of reduction is approximately 2 m.

### Thesis data

In order to monitor the dynamic changes of the groundwater level, 30 observation wells (2001–2013) are set up in the area of 550 km^2^ (the plain area and the piedmont area). Quaternary Stata in the piedmont plain is taken as the studying object. Water level measuring tape is used to monitor groundwater level and the observation is made every 5 days (i.e. observations are made on 1st, 6th, 11th, 21th, 26th days of each month). The land use data is collected from the aerial map in 2005, the resolution is 1 m.

**Fig. 2:**
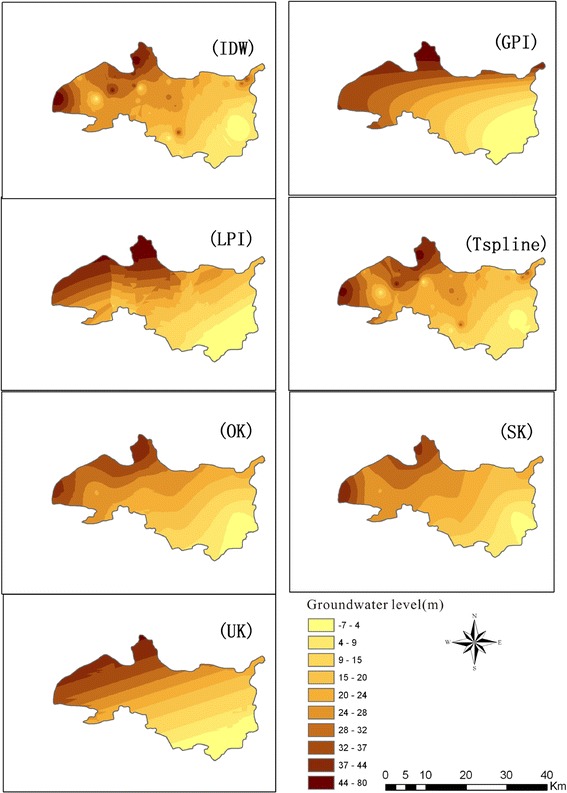
Interpolation effect of groundwater level in Beijing piedmont plain through the seven interpolation models in 2013

To understand the character of the datasets, observed values of 30 observation wells for 2013 have been statistically analysed (Fig. 2). It was found that the groundwater level ranges from −7.8 to 61.4 m, the mean value is 26.4 m, and the standard deviation of the data is 17.8 m. The kurtosis is 2.66 and the skewness is 0.01. The dataset follows a normal distribution and the Kriging interpolation can be applied. In order to obtain the distribution characteristics of groundwater level, the selection of model parameters shall meet the principle that the error is minimum and the parameters are used to verify the model. The selected parameters are shown in Table 1.

**Table 1:** Parameters using in the interpolation model

Interpolation model	Parameter P or smooth coefficient^a^	Maximum number of prediction points within the search radius	Minimum number of prediction points within the search radius	Orientation angle^b^
IDW	1.1102	16	9	0
GPI	2	–	–	–
LPI	1	14	11	0
TSPLINE	1	15	10	0
OK	0.12	13	9	45
SK	0.138	5	2	0
UK	1	30	20	45

^a^The order that is used to calculate exponential value in the weighting formula or optimal fitting polynomial

^b^Search direction angle within the search radius

### Research method

The study method is mainly composed of the following steps:

1. *Construction of basic information database* ArcGIS data management is used to obtain the locator and attribute data of groundwater level, land use for each observation well and use them as the basic data for calculation and analysis with geo-statistical module.
2. *Evaluation of different interpolation model* Inverse distance interpolation (IDW), GPI, LPI, TSPLINE, OK, SK and UK are applied to establish the groundwater level model, mean errors, root-mean-square errors, coefficient of determinations and absolute errors are calculated for each method to select the optimal model. The computational formulas are as follows (Nikroo et al. 2010; Sun et al. 2009):
  1. Mean error
      1 $$ME = \frac{1}{n}\left( {Z_{i} - \hat{Z}_{i} } \right)$$
      where $\hat{Z}_{\text{i}}$ is the estimated value, *Z*_*i*_ is the measured value at sampling point i $({\text{i}} = 1, \ldots ,{\text{n}})$, n is the number of values used for the estimation.
  2. Root-mean-square error
      2 $$RMSE = \sqrt {\frac{1}{n}\left( {Z_{i} - \hat{Z}_{i} } \right)^{2} }$$
      where $\hat{Z}_{\text{i}}$ is the estimated value, *Z*_*i*_ is the measured value at sampling point i $({\text{i}} = 1, \ldots ,{\text{n}})$, n is the number of parameters selected by the empirical formula; n is the number of values used for the estimation.
  3. Coefficient of determination
      3 $$R^{2} = \frac{{\left[ {\sum\nolimits_{i = 1}^{n} {\left( {P_{i} - P_{ave} } \right)\left( {Q_{i} - Q_{ave} } \right)} } \right]^{2} }}{{\sum\nolimits_{i = 1}^{n} {\left( {P_{i} - P_{ave} } \right)^{2} \sum\nolimits_{i = 1}^{n} {\left( {Q_{i} - Q_{ave} } \right)}^{2}} }}$$
      The coefficient of determination R^2^ is used to measure the correlation between the predicted value and the measured value. *P*_*ave*_ is the averaged estimated value, *Q*_*ave*_ is the averaged measured value; and n is the number of values used for estimation.
3. Semivariogram, also known as semi-variogram, is a unique function of geostatistical analysis. It can be used to describe the spatial variability of groundwater levels. Assume that the mean of the random function is stable and the value is only related to the distance between samples, semivariogram r(h) may be defined as half the incremental variance of random function Z(x).
  4 $$r(h) = \frac{1}{2}E\left[ {Z(x) - Z(x + h)} \right]^{2}$$
  where *r*(*h*) is the semivariogram, *Z*(*x*) is the random function, *h* is the distance between samples.
  A spherical model of the tested semivariogram models was fitted to the experimental semivariograms. The spherical model is defined by the following equation:
    5 $$\rho (h) = \left\{ {\begin{array}{*{20}l} 0 \hfill &\quad {x = 0} \hfill \\ {{\text{H}}_{0S} + {\text{H}}_{S} \left( {\frac{{3{\text{h}}}}{{2{\text{a}}}} - \frac{{h^{3} }}{{2a^{2} }}} \right)} \hfill &\quad {0 < x \le b} \hfill \\ {{\text{H}}_{0S} + {\text{H}}_{S} } \hfill &\quad {x > b} \hfill \\ \end{array} } \right.$$
    where *H*_0*S*_ is the nugget value arising from random components such as measurement error and physical factors, *H*_*S*_ is the structural variance arising from spatial autocorrelation, H_0S_ + *H*_*S*_ is the sill, and *b* is the distance at which the semivariogram equals 95 % of its sill variance.
4. Spatial variability characteristics, is represented by the nugget to sill radio. The nugget value represents the variability while the sill value represents the overall variability inside the variables. The nugget to sill radio shall be between 0 and 1. When the nugget effect is <0.25, the considered strongly spatially dependent; the variable is considered moderately spatially dependent as the nugget effect is between 0.25 and 0.75, while the nugget effect is >0.75, the variable is considered weakly spatially dependent (Ghazi et al. 2014).
5. Analysis of spatial distribution pattern: based on the hydrogeological conditions and different land use, the spatial variability pattern of typical partitions in the piedmont plain are analyzed, the effect of human activities on spatial variability of groundwater level is also discussed.

## Results and discussion

### Model parameter selection and verification

In order to obtain the distribution characteristics of groundwater level, the selection of model parameters shall meet the principle that the mean errors and root-mean-square errors are close to 0 and 1, respectively. The parameters occur in the Geostatistical Analyst Model of Arcmap 10.3, which are used to verify the model and approach the best interpolation effect for each method. The selected parameters are shown in Table 1.

### Interpolation calculation results

The cross validation method is a statistical analysis method used to verify the accuracy of interpolation model, the basic idea is to classify the original dataset into the train set and the validation set. The validation set is used to test the model obtained from the training set, which is the indicators to evaluate the accuracy of the model. The error being the minimum is the evaluation criteria for best-fit interpolation model (Dashtpagerdi et al. 2013).

**Fig. 3:**
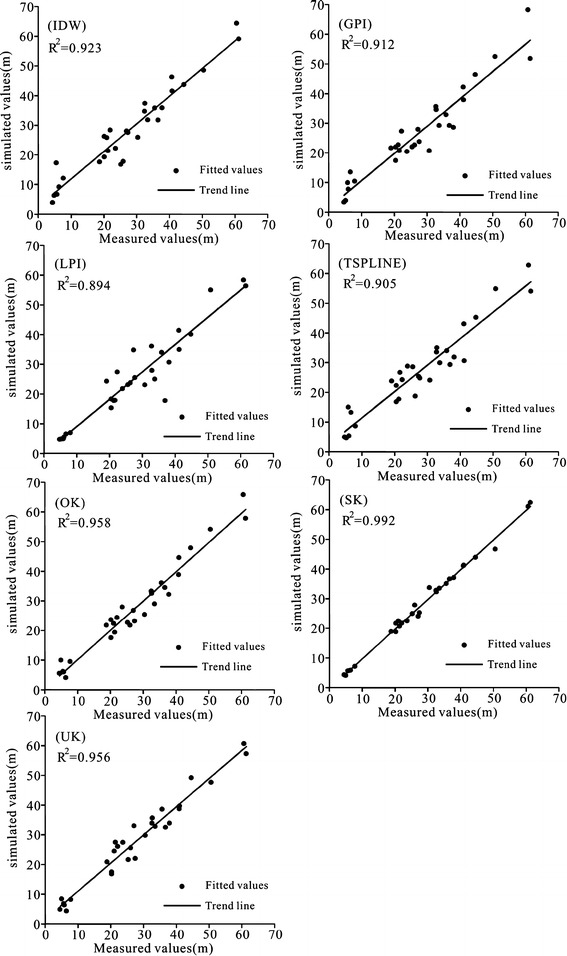
Comparison of simulated and measured groundwater levels

Table 2 is the cross validation results of the groundwater observation data in the last 5 years (2009–2013). Take the year 2013 as an example, the root-mean-square error sorting is SK < UK < OK < IDW < LPI < TSPLINE < GPI; the mean error sorting is TSPLINE = SK < UK < OK < LPI < IDW < GPI, the R^2^ sorting is GPI < TSPLINE < LPI < IDW < UK < OK < SK. By combining the results from different methods, GPI is the worse-fit model, which is based on the global interpolation and is not precise interpolation. With the increase of number of times, the interpolation effect is better but the complexity and error also increase (Johnson et al. 2001a). LPI interpolation method is suitable for local spatial interpolation and is of high accuracy in simulating short-term variability. With regard to analysis of groundwater level for a period of 10 years, the prediction results fluctuate greatly and doesn’t fit well (Rajagopalan and Lall 1998). Because of extreme points, the mean error and coefficient of determination of IDW model as well as TSPLINE model are relatively high and the simulation accuracy is poor. The error results of three Kriging interpolation methods (OK, UK and SK) are close to each other. In combination with Table 2 and Fig. 3, the geostatistical interpolation method, including original Kriging, simple Kriging and UK interpolaiton method, can obtain good simulation results, and R^2^ of simple Kriging is 0.99, which is considerd as the optimal method for interpolating groundwater level.

**Table 2:** The statistic of errors in the process of groundwater interpolation in the last 5 years (2009–2013)

Year	Errors	Methods						
2013	Interpolation model	IDW	GPI	LPI	TSPLINE	OK	SK	UK
	Mean error	0.17	−0.73	0.14	0.02	−0.04	0.02	0.03
	Root-mean-square (m)	0.89	1.46	1.07	1.24	0.26	0.11	0.24
	R^2^	0.92	0.89	0.91	0.90	0.96	0.99	0.96
2012	Interpolation model	IDW	GPI	LPI	TSPLINE	OK	SK	UK
	Mean error	0.11	0.79	−0.08	0.04	−0.10	0.03	−0.09
	Root-mean-square (m)	0.92	1.49	1.10	1.27	0.29	0.04	0.47
	R^2^	0.88	0.85	0.87	0.86	0.92	0.95	0.92
2011	Interpolation model	IDW	GPI	LPI	TSPLINE	OK	SK	UK
	Mean error	0.19	−0.71	0.16	−0.04	0.02	−0.01	0.01
	Root-mean-square (m)	0.17	0.72	0.14	0.02	0.04	0.02	0.03
	R^2^	0.91	0.88	0.90	0.89	0.95	0.98	0.95
2010	Interpolation model	IDW	GPI	LPI	TSPLINE	OK	SK	UK
	Mean error	0.19	−0.82	0.18	0.02	0.04	0.04	0.03
	Root-mean-square (m)	0.85	1.40	1.03	1.19	0.25	0.18	0.42
	R^2^	0.90	0.87	0.89	0.88	0.94	0.97	0.94
2009	Interpolation model	IDW	GPI	LPI	TSPLINE	OK	SK	UK
	Mean error	0.14	−0.77	−0.06	0.07	−0.07	0.05	−0.06
	Root-mean-square (m)	0.90	1.45	1.08	1.24	0.30	0.23	0.47
	R^2^	0.88	0.86	0.89	0.89	0.92	0.98	0.95

### Interpolation effect comparison among different models

Figure 2 shows the interpolation effect of groundwater level in Beijing piedmont plain from seven interpolation model for 2013. In order to evaluate the simulation results, the “buphthalmos” phenomenon is introduced (Yang et al. 2011). Which is caused by some extreme values in the processing of interpolation, it means the interpolation effect is not good.

As can be seen from Fig. 2, uneven distribution of observation points and the existence of some extreme value points leads to the “buphthalmos” phenomena for IDW and LPI methods (Rajagopalan and Lall 1998) and the interpolation effect is poor; as a function form of radial basis function (RBF) interpolation model, TSPLINE is suitable for establishing a smooth surface with a large amount of data and the interpolation effect is good for undulating surface. However, it is not suitable for the situation where the short-term variability is high, sample data errors and uncertainties exist. Therefore, the smoothness of the model is poor. GPI considers the global variation trend during the interpolation process and the smoothness is high, which is suitable for spatial variability interpolation whose variability is slow. However, GPI ignores the local variability and its fitting effect of the first-order model is not good (Johnston et al. 2001b). With the increase of order, it is difficult to explain the precise physical meaning (Apaydin et al. 2004).

As the unbiased optimal interpolation method, Kriging method combines the effects of distance parameters and direction parameters, it represents the spatially continuous and irregular change of variables. Therefore, the interpolation effect of Kriging model is better than others in theory. From the perspective of interpolation results, the SK interpolation is more approximate to the real situation and the groundwater surface looks smooth, which means the interpolation effect is good. Based on the above analysis, SK interpolation method is evaluated as the optimal interpolation model.

### Spatial variability analysis of groundwater level

The SK interpolation is used to analyze the spatial variability characteristics of groundwater level between 2001 and 2013 to understand the distribution pattern and variability characteristics of the groundwater level. The spatial variability of the groundwater level is affected by both the natural factors and human factors. The nugget effect reflects the spatial correlation characteristics of the groundwater level (Ahmadi and Sedghamiz 2007; Desbarats et al. 2002). Factors such as precipitation, topographic inequality, aquifer lithology and different hydrogeological units will increase the spatial correlation of groundwater level, while factors such as large-scale construction of water conservancy establishments and human exploitation will decrease the spatial correlation.

As can be seen from Table 3, the nugget effect of the groundwater level during 2001 and 2013 is gradually increasing, which means the spatial correlation decreases and the effect of human activities increases continuously. During 2001 and 2005, the nugget effect of the groundwater level is between 0.25 and 0.75, which means the groundwater level is affected by both natural factors and human factors, while natural factors have more significance than human factors. In 2006 and 2007, the spatial correlation decreases as a result of less precipitation, the nugget effect increases to 0.65. The precipitation in 2008 is abundant and the source of supply is sufficient, while Olympic Games in Beijing leads to a large-scale development in urban, the establish of Machikou emergency water sources and the expansion of Nankou water plant increase the consumption of the groundwater. The precipitation recharge cannot meet the shortfall of the groundwater and the storage resource is used. The groundwater level is affected mostly by human exploitation and the nugget effect increases to 0.73, the spatial correlation of groundwater level is weakened. During 2009 and 2013, the excessive exploration of the groundwater is controlled to some extent. The nugget effect begins to decrease while the spatial correlation increases.

**Table 3:** Semi-variance function model parameters of groundwater level (2001–2013)

Year	2001	2002	2003	2004	2005	2006	2007	2008	2009	2010	2011	2012	2013
Nugget value	79.3	83.5	90.3	81.6	83.8	92.3	116.9	129.6	106.6	97.2	99.2	103.1	107.2
Partial sill value	135.6	124.9	115.8	105.1	103.9	78.3	63.0	48.9	72.3	87.2	85.6	82.1	78.2
Sill value	214.9	208.4	206.1	186.7	187.7	170.6	179.9	178.5	178.9	184.4	184.8	185.2	185.4
Nugget effect	0.37	0.40	0.44	0.44	0.45	0.54	0.65	0.73	0.60	0.53	0.54	0.56	0.58

### Analysis of spatial distribution characteristics

**Fig. 4:**
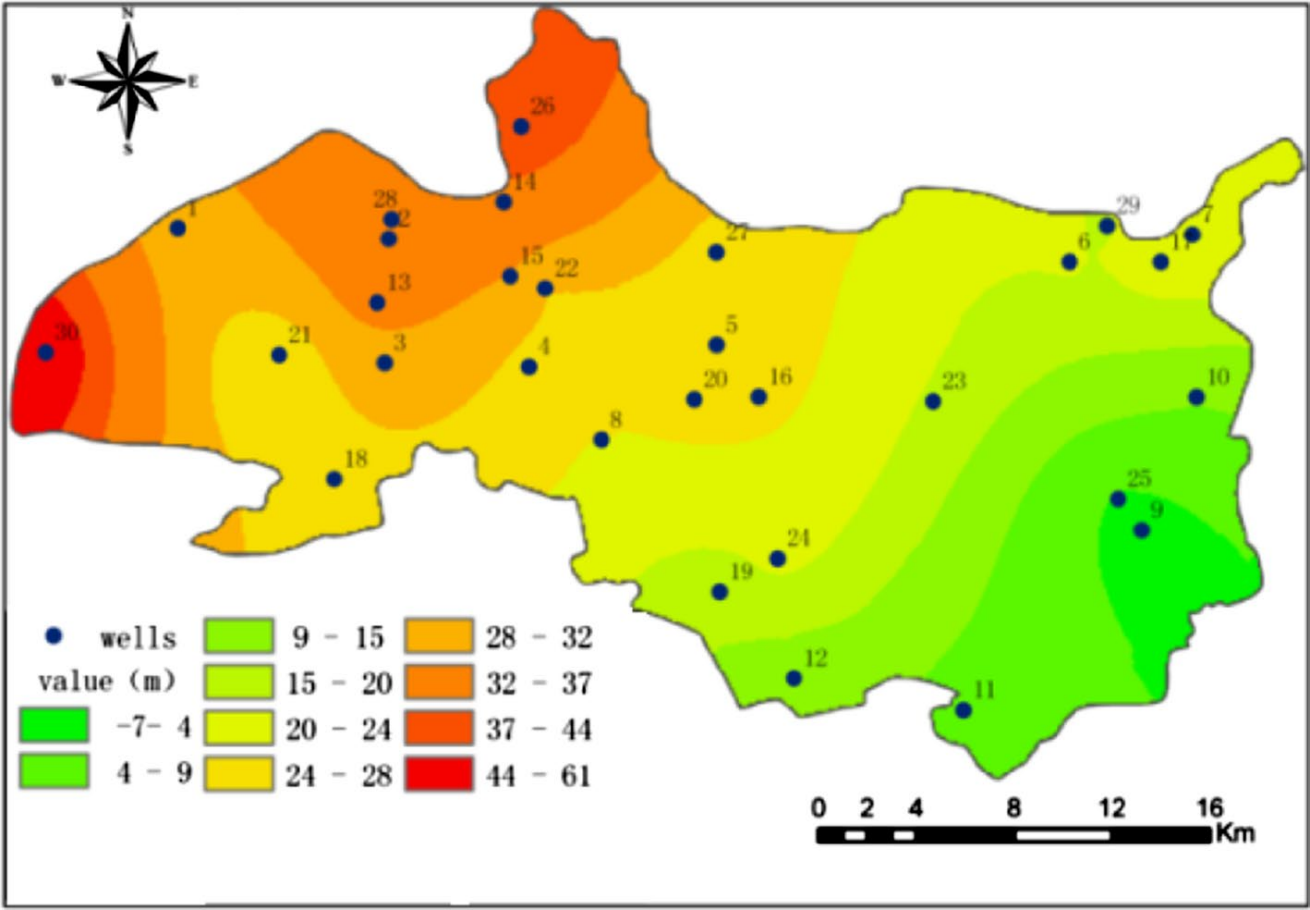
Groundwater level distribution in 2013

Figure 4 shows that flow filed of groundwater flows from the recharge area in the northwest to the plain area in the southeast for 2013. The groundwater level is between −7 and 61 m, the average groundwater level is 30 m. The spatial variability of the groundwater level in the middle part of the alluvial–proluvial fan is relatively higher than in the top and bottom.

**Fig. 5:**
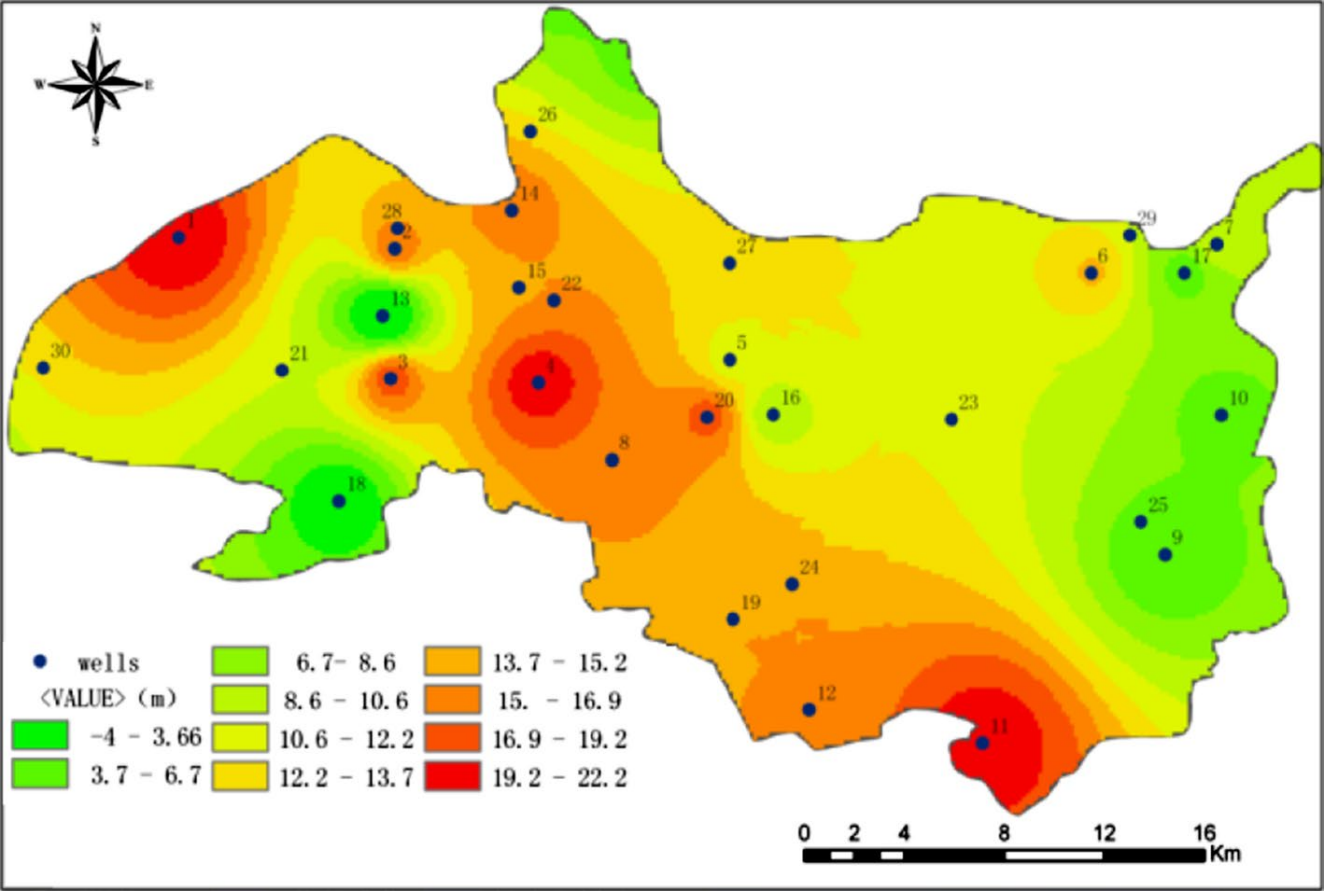
Groundwater level drawdown during 2001 and 2013

During 2001 and 2013, the groundwater level declines generally and the maximum drawdown is 22 m. The average reduction speed is 2 m annually (Fig. 5). The piedmont area is located in the top of alluvial–proluvial fan, the aquifer is the sand and gravel of coarse particles with good vertical infiltration and sufficient precipitation recharge. At the same time, groundwater level is deep, which is not conductive for exploitation, so the spatial variability of the groundwater level in the top area is small; the areas whose drawdown is between 16 and 22 m are mainly distributed in the middle and upper part of the alluvial–proluvial fan, the aquifer is of many layers of sand and the groundwater level is shallow, which make it the concentrated exploitation regions, Nankou water plant and Xiguan water plant are located in the north with annual water supply capacity of 3,000,000 m^3^. Machikou (No. 4) emergency water sources supply water for new urban district. In the vicinity, the groundwater drawdown is more than 20 m. Shahe reservoir and Shahe water plant are located in the south, the southeast area is the urban water supply partition. Due to speeding up of urbanization in recent years and large exploitation quantity of the groundwater, the spatial variability is very high in the middle and upper part of the alluvial–proluvial fan; the spatial variability is very small in the east part of the plain, which is located in the lower part of the alluvial–proluvial fan and the aqueous layer is not continuous. The cone of groundwater depression caused by over-exploration cannot be effectively recharged. In recent years the prohibition of exploitation and restriction on exploitation are enforced, which increases the groundwater level.

**Fig. 6:**
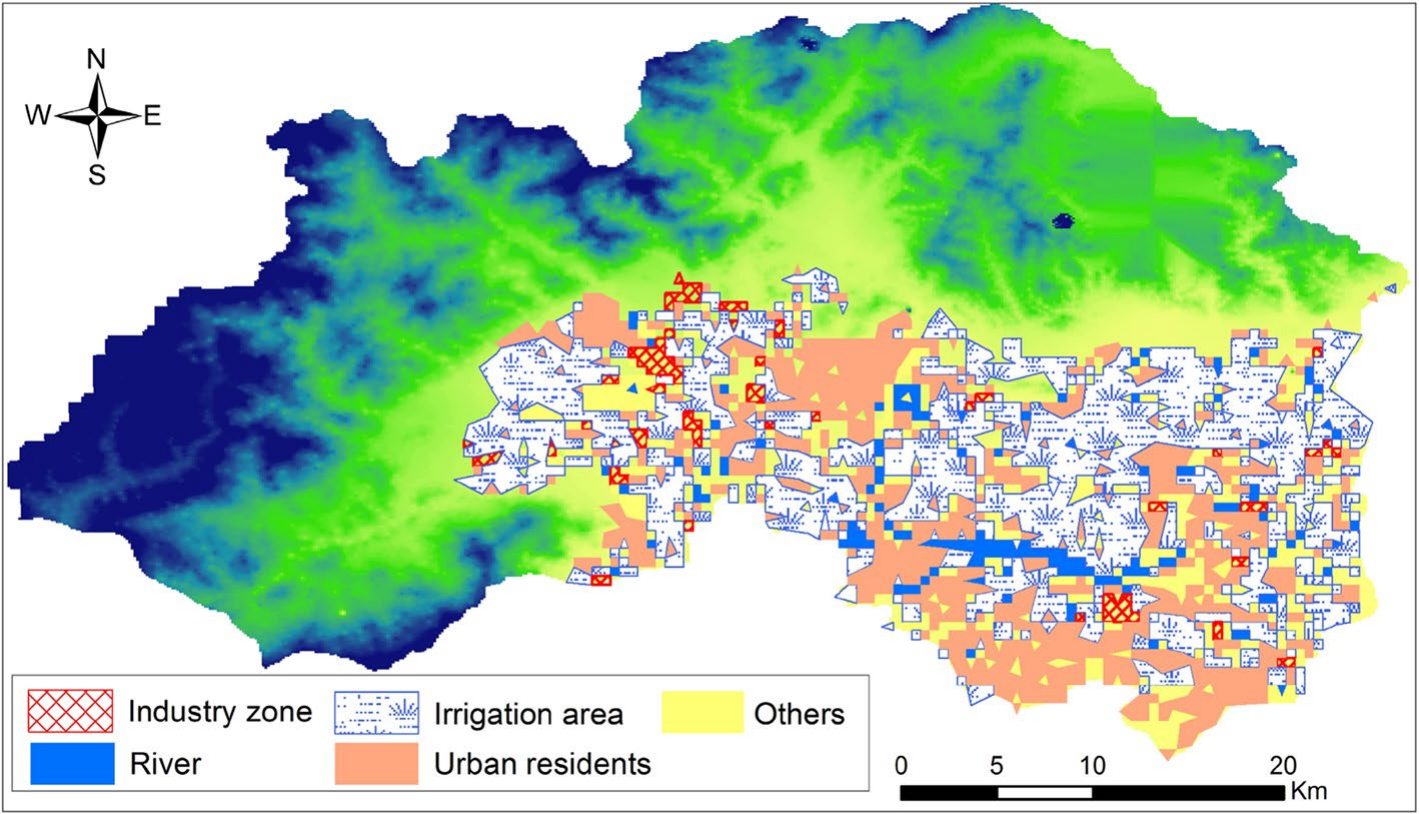
Land use types of the study area

In order to further study the temporal variation of groundwater level, groundwater level reduction rate of the typical regions for different land use was analyzed (Table 4). The results show that the groundwater level reduction speeds for the residential and industrial area are 0.77 and 0.65 m/a respectively during 2001 and 2006 (Fig. 6). Since the year 2000, the agricultural water proportion has decreased and the industrial water proportion and the domestic water proportion have increased. Therefore, the groundwater level reduction speeds are high in the residential area and industrial area. At the same time, the enforcement of water-saving irrigation causes the agricultural water quantity to decrease continuously. The reduction speed of the groundwater level is only 0.25 m/a; for the upstream region of the river, the groundwater level reduction rate is not high because of the surface water infiltration recharge and sufficient piedmont lateral flow recharge.

**Table 4:** The average annual decline rate of groundwater level for observation wells

Period	Average annual decline rate	Land use			
		Agricultural area	Residential area	Industrial area	River area
2001–2006	0.51	0.35	0.77	0.65	0.25
2007–2013	2.06	0.65	1.28	2.34	3.95

Because of over-exploitation, the annual average reduction speed during 2007 and 2013 is higher than that during 2001 and 2006. Under the same land use, the change of exploitation way of the groundwater will cause the change of reduction speed of the groundwater level (Choi et al. 2012). Due to the rapid development of the urban area and increase of exploitation amount of the groundwater year by year, the groundwater level during 2006 and 2013 presents an overall downward trend. In the residential area and industrial area, the exploitation quantity of the groundwater is high and the groundwater level reduction speed increases significantly; the agriculture area is mainly distributed in the middle and lower plain area of the alluvial–proluvial fan. The exploitation quantity of the groundwater is of small amount and the reduction speed is small; in the middle and lower river area of the alluvial–proluvial fan, the reduction speed of the groundwater level increases significantly. The reasons are as follows: the severe shortage of the surface water decreases the lateral infiltration recharge quantity; excessive exploitation causes the lateral recharge rate of the piedmont groundwater to be smaller than the groundwater level reduction speed in the river area; the complete lining of the Jingmi diversion canal in the central part of the plain area also aggravates the situation that the groundwater cannot be recharged in time.

## Conclusion

1. This paper is based on the ArcGIS geo-statistical module and semi-variable function model to analyze the spatial variability of the groundwater level, compare the simulation accuracies and prediction effects of seven interpolation models and select SK interpolation as the optimal interpolation model for the piedmont plain in Beijing. The results show that the SK interpolation can make unbiased optimal estimation of unknown points while ensuring the local accuracy.
2. The spatial variability of the groundwater level is closely related to the hydrogeological units and land use. Owing to the excessive exploitation, the spatial variability of the groundwater level for the top and lower part of the alluvial–proluvial fan is small; the spatial variability of the groundwater level for the middle and upper part of the alluvial–proluvial fan is high; In the residential area, industrial area and river area, the reduction speed of the groundwater level is high; the establishment of new town causes the arable land to decrease and water-saving irrigation decreases the agricultural water quantity, and the reduction rate of the groundwater level is small in the agricultural area.
3. The simple Kriging method is applicable for the interpolation of groundwater level in piedmont plain area and the geostatistical interpolation models are of significance in studying the spatial and time variability of the groundwater level. It can also effectively optimize the exploited well distribution and slow down the decline rate of groundwater level.
